# How Early Life Stress Impact Maternal Care: A Systematic Review of Rodent Studies

**DOI:** 10.3389/fnbeh.2019.00197

**Published:** 2019-08-28

**Authors:** Rodrigo Orso, Kerstin Camile Creutzberg, Luis Eduardo Wearick-Silva, Thiago Wendt Viola, Saulo Gantes Tractenberg, Fernando Benetti, Rodrigo Grassi-Oliveira

**Affiliations:** ^1^Developmental Cognitive Neuroscience Lab (DCNL), School of Medicine, Pontifical Catholic University of Rio Grande do Sul (PUCRS), Porto Alegre, Brazil; ^2^School of Medicine, Brain Institute (Instituto do Cérebro), Pontifical Catholic University of Rio Grande do Sul (PUCRS), Porto Alegre, Brazil; ^3^Laboratório de Neurofisiologia Cognitiva e do Desenvolvimento, Department of Physiology, Instituto de Ciências Básicas da Saúde, Federal University of Rio Grande do Sul (UFRGS), Porto Alegre, Brazil

**Keywords:** early life stress, maternal care, maternal behavior, rodent, systematic review, postnatal stress

## Abstract

**Background:** Maternal care refers to the behavior performed by the dam to nourish and protect her litter during its early development. Frequent and high-quality performance of such maternal behaviors is critical for the neurodevelopment of the pups. Maternal exposure to stress during early development can impair maternal care and amplify the deleterious effects of poor maternal caregiving and neglect. As such, a thorough understanding of the effects caused by several models of early life stress on maternal care may yield more insights into the relationship between stress and maternal behavior.

**Methods:** A systematic review was performed to identify and address the effects of early life stress on maternal behavior. The search was conducted using three online databases: PUBMED, Embase, and Web of Science. To provide clear evidence of the impact of stress on maternal care, in every study, the stress group was always compared to a control group. Outcomes were categorized into eight different behaviors: (1) licking/grooming; (2) arched-back nursing; (3) blanket-nursing/passive nursing; (4) nest building; (5) contact with pups; (6) harmful/adverse caregiving; (7) no contact; (8) nest exits. Additionally, the methodological quality of the studies was evaluated.

**Results:** A total of 12 different early life stress protocols were identified from the 56 studies included in this systematic review. Our data demonstrate that different stress models can promote specific maternal patterns of behavior. Regarding the maternal separation protocol, we observed an overall increase in nursing and licking/grooming behaviors, which are essential for pup development. An increase in the number of nest exits, which represents a fragmentation of maternal care, was observed in the limited bedding protocol, but the total amount of maternal care appears to remain similar between groups.

**Conclusions:** Each stress protocol has unique characteristics that increase the difficulty of rendering comparisons of maternal behavior. The increase in maternal care observed in the maternal separation protocol may be an attempt to overcompensate for the time off-nest. Fragmented maternal care is a key component of the limited bedding protocol. Moreover, the methodological approaches to evaluate maternal behavior, such as time, duration, and behavior type should be more homogeneous across studies.

## Introduction

Maternal caregiving plays a key role in childhood development (Cirulli et al., 2003; Orso et al., 2017). Such behavior is classified as any interaction prompted by the dam to nourish and protect the pups during the first weeks of their development. Besides providing nutrition and regulating temperature, it ensures the necessary stimuli for neurodevelopment is essential (Champagne et al., 2003). More pronounced maternal responses start appearing in preparation for birth, as expectant dams demonstrate increased levels of aggression toward intruders and display nest building behavior to protect and prepare the environment for the litter. After birth, the dam engages in licking and nursing the pups, which is necessary for pup development (Bridges, 2015). Besides the expected adaptations in behavioral patterns, the dams also manifest hormonal and neurochemical alterations. Endocrine changes that occur during birth, such as increased levels of circulating estradiol, progesterone, and lactogenic hormones, are associated with spontaneous maternal care after birth (Morishige et al., 1973; Siegel and Rosenblatt, 1975). Moreover, neuropeptides and neurotransmitters, such as oxytocin, arginine vasopressin, dopamine, and serotonin, are highly associated with the necessary stimuli for the development of maternal behavior (Pedersen et al., 1982; Numan et al., 2005).

The quality and frequency of maternal care have been shown to critically affect the maturation of brain, behavior, cognitive, and emotional systems of the offspring of several mammalian species (Bath et al., 2016; Guardini et al., 2017). In humans, longitudinal studies have documented that poor maternal care is a well-established risk factor for a myriad of neuropsychiatric diseases in offspring (Shin et al., 2013; Sacks et al., 2017). For instance, childhood exposure to maternal neglect is associated with increased risk of suicide attempts (odds ratio-OR = 1.85), drug abuse (OR = 1.36), attention-deficit/hyperactivity disorder (OR = 2.02), and for the development of depressive (OR = 2.11), anxiety (OR = 1.82), and bipolar (OR = 2.02) disorders later in life (Norman et al., 2012; Agnew-Blais and Danese, 2016; Stern et al., 2018). Given the deleterious effects of poor maternal caregiving and neglect, knowledge of the neurobiological consequences underlying these behavioral phenotypes in the offspring is essential. Therefore, preclinical animal models have been consistently used to pursue a translational approach, aimed at revealing the molecular signature of altered brain development trajectories attributable to variations in maternal caregiving (Meaney and Szyf, 2005).

In basic neuroscience, rats, and mice are definitely the most commonly used species, and compelling evidence has shown stable individual differences in rodent maternal care behaviors over the first weeks of lactation, notwithstanding any experimental manipulation (Meaney, 2010). Analysis of the relationship between mother and pups during the first weeks of postnatal development has been constantly deployed as a predictor of offspring individual differences later in life (Weaver et al., 2004). To address that, basic maternal behaviors, such as frequency of nursing, as well as licking and time spent with pups, are recorded over the course of several days and at different daily periods, including morning, afternoon, and evening (Champagne et al., 2007). Besides the studies that assemble maternal care data through the observation of natural home-cage behaviors, some studies utilize specific maternal responsivity tests. For example, Murgatroyd and Nephew (2013) analyzed maternal behavior after removing the pups from the home cage for 30 min, and then evaluated maternal responsivity after re-introducing the pups.

Evidence indicates that pups raised by a dam exhibiting low levels of maternal care can present with neurobiological consequences (e.g., structural, functional, biochemical, and molecular alterations), such as altered hypothalamic–pituitary–adrenal (HPA) axis functioning (Champagne et al., 2003; Weaver et al., 2004; Champagne and Meaney, 2006). Moreover, such pups demonstrate higher levels of cocaine/alcohol consumption, as well as anxiety and depressive-like behaviors, resembling the phenotypes found in humans exposed to poor maternal caregiving (Francis and Kuhar, 2008; Meaney, 2010). Recent evidence suggests that maternal care disruption also impacts epigenetic processes, such as DNA methylation, histone modification, and microRNA expression, which can be associated with long term behavioral outcomes (Curley and Champagne, 2016).

Despite this naturally occurring variability in maternal care, some preclinical studies often aim to manipulate the interaction between dam-pups to investigate the effects of reduced maternal caregiving on multiple outcomes (Holmes et al., 2005; Roman and Nylander, 2005; Nylander and Roman, 2013). Several types of postnatal manipulations have been proposed to affect the rodent dam-pup relationship and generate a stressful condition early in life, including models that involve handling of neonatal pups (Plotsky and Meaney, 1993), maternal separation (MS) (Millstein and Holmes, 2007), and dam-pup exposure to reduced nesting material (Rice et al., 2008). Such studies have documented that maternal caregiving can be significantly altered by stressful experimental experiences, suggesting that the long-lasting consequences of rodent models of early life stress are attributable, at least in part, to altered maternal care behavior (Tractenberg et al., 2016).

It is important to highlight that the data available in the literature are conflicting and that no consensus exists on how rodent models of maternal caregiving may be altered by stressful environmental experiences. This may be explained by the wide variety of early-life stress protocols and the modes of analyzing maternal behavior in the neonatal period. Among the most studied early-life stress protocol is MS, which is a well-established model to induce long-lasting behavioral, molecular and neuroanatomical alterations (Grassi-Oliveira et al., 2016). This model consists of repeated separations of the litter from the dam across several days. Regarding the different models of maternal separation, they can be distinguished according to the duration period the pups remain away from the home cage and dam. According to Tractenberg et al. (2016), a short period of time, ranging from 10 to 60 min, is classified as brief maternal separation (BMS), but if the duration exceeds the 60 to 480 min range, it is classified as prolonged maternal separation (PMS). It is important to consider the time factor, as some studies report that brief periods of separation do not impair development of the pups, but that prolonged separation may engender an inadequate environment for neurodevelopment (Macrì et al., 2008; Azevedo et al., 2010). The MS protocol has several variations, not only in terms of the duration of the protocol, but also in the daily duration, as well as temperature variations, whether litters are isolated as a whole or individual pups and type of separation. The comparison group in maternal separation studies are another important factor, considering the amount of handling procedures required for this protocol. Some studies ended up using non-handled controls, brief handling (15 min of separation) or AFR litters (animal facility-reared, standard cage-cleaning procedures). Given all these variations, comparing the results from different laboratories and rendering an interpretation of that comparison becomes highly complex. There are other protocols of postnatal environmental manipulations available in the literature that, relative to MS, dramatically reduce the amount of manipulations required. The limited bedding (LB) protocol, where mothers are provided with reduced nesting and bedding material, results in changes in maternal behavior and fragmented maternal care (Brunson et al., 2005; Roth and Sullivan, 2005; Ivy et al., 2008). Furthermore, the introduction of a new environment, without the necessary material to build an adequate nest, can cause the dam to manifest potentially harmful behaviors, which can have a profound impact on the development of the pups (Walker et al., 2017).

Given the wide variety of alterations in maternal care behavior that may result from neonatal stress exposure, researchers may struggle to determine the basis for selecting and designing an experiment that has the ultimate goal of affecting maternal caregiving through neonatal environmental stress. In addition, as there is no consensus on the procedures of maternal behavior data collection, such as the frequency and periods of observations, researchers also face uncertainties in these areas as they endeavor to establish their protocol for behavioral analysis. Systematic reviews of animal studies, including rodent models, are needed, because they provide reliable information on selecting the appropriate methods and procedures, according to the goals of the researchers (Tractenberg et al., 2016). The question that underpinned this systematic review was: *How do different early life stress protocols affect maternal care?* The main goal of this study was to systematically review animal models of early-life stress in terms of its impact on maternal behavior. In addition, given the aforementioned methodological variations and their effects on the outcome, we sought to provide detailed methodological approaches of the selected studies.

## Methods

### Search Strategy

**Figure 1:**
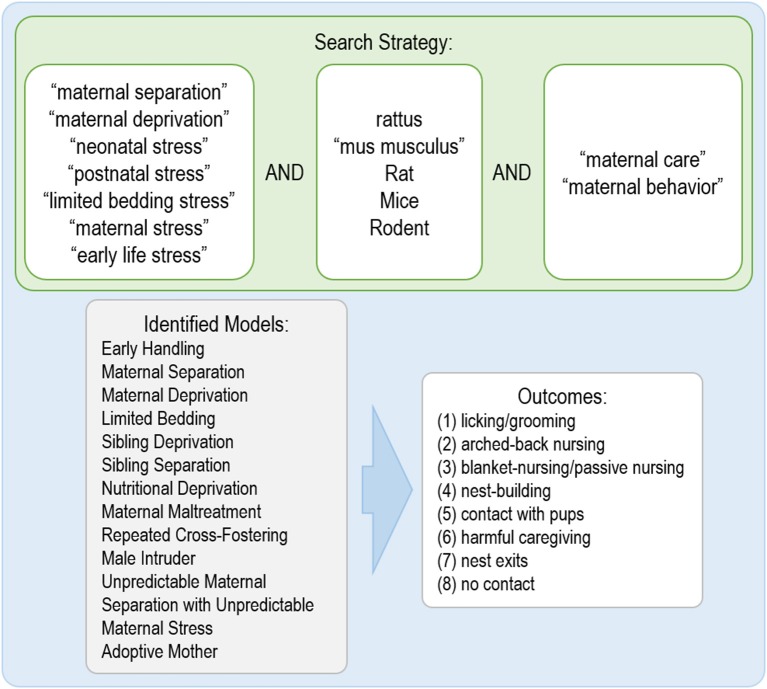
Terms used for the search, identified models in the studies and behavioral outcomes evaluated in the systematic review.

The search was conducted using three online databases, PUBMED, Embase, and Web of Science, using the following terms: [“maternal care” OR “maternal behavior”] AND [Rattus OR “mus musculus” OR rat OR mice OR rodent] AND [“maternal separation” OR “maternal deprivation” OR “neonatal stress” OR “postnatal stress” OR “limited bedding stress” OR “maternal stress” OR “early life stress”] (Figure 1). This systematic review followed the recommendations of Cochrane for developing a search strategy (Cochrane Infectious Diseases Group, 2007) and the Systematic Review Center for Laboratory Animal Experimentation (SYRCLE) (Hooijmans et al., 2014). The search was performed on August 1st, 2018.

### Eligibility

After identification of duplicate entries among searches and databases, two authors (RO, KCC) reviewed the abstracts and full reports of original studies. Five exclusion criteria were applied, following a hierarchical order:

- The study was not an empirical;
- The study did not use mice or rats;
- The study did not use an early life stress protocol;
- The study did not involve maternal care observation under undisturbed home-cage conditions;
- The study used only transgenic or knockout animals.

Any discordance during this process was discussed with a senior scientist (RGO) to reach a final agreement about inclusion or exclusion of a given study. Furthermore, to search for any additional study, the reference list of all included papers was manually analyzed. For a detailed flow chart of the systematic review, see Supplementary Figure 1.

### Data Extraction and Methodological Assessment

From each study, the following data was extracted by two independent authors (KCC, RO): authors, publication year, strain, litter size control method, existence of cross fostering, stress period, stress model, stress duration, maternal behavior observation period, duration of maternal behavior observation, outcome variables, and methodological quality. The summary of the descriptive data obtained from all eligible studies is shown in Supplementary Table 1 (rat studies) and Supplementary Table 2 (mice studies).

The next step was to summarize the findings, regarding the effects of all early life stress protocols on maternal behavior. The studies were manually examined by two independent investigators (KCC and RO). To provide clear evidence of the stress impact on maternal behavior, the stress group was always compared to a control group (no stress exposure), as several studies had more than one stress group. The effects of early life stress on maternal behavior were classified as increased, decreased or no alteration on maternal caregiving (Details in Supplementary Tables 1, 2).

To determine not only the findings for each study, but also the methodological quality of the papers included in this review, all studies were evaluated using an adapted version, developed by Tractenberg et al. (2016), of the Gold Standard Publication checklist (GSPC) (Hooijmans et al., 2011) and the ARRIVE Guidelines for Guidelines for Reporting Animal Research (Kilkenny et al., 2010). Two authors (RO and KCC) independently performed the checklist analysis, using a 28-item checklist method: 0.5 points were recorded when a specific data was presented in the article, and 0 points when the information did not appear in the article. The minimum score a study could achieve was 0, and the maximum score was 14. This checklist accounted for methodological aspects that may influence the overall maternal behavior. Some examples of items included in the checklist are as follows: housing conditions, litter control, maternal behavior blinding, early life stress time and duration, maternal behavior evaluation time and duration and outcomes description. Data corresponding to the methodological quality of the studies are presented in Supplementary Figure 2 for rat studies and Supplementary Figure 3 for mice studies.

### Maternal Behavior Characteristics

**Figure 2:**
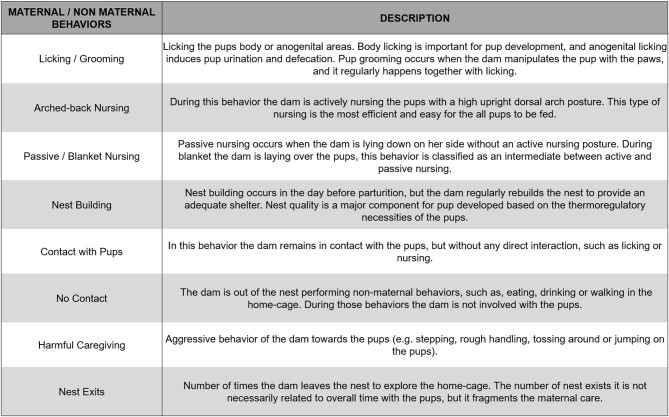
Description of the behavioral categories included in the systematic review.

An analysis of the literature was performed by two authors (KCC and RO) to provide conceptual information regarding specific types of maternal behaviors. The characteristics of each behavior are based on the dam's behavior and interaction with pups. To standardize the interpretation of each interaction, we divided the maternal behaviors into eight categories, based on the studies included in this review: (1) licking/grooming; (2) arched-back nursing; (3) blanket-nursing/passive nursing; (4) nest building; (5) contact with pups; (6) harmful/adverse caregiving; (7) no contact; (8) nest exits. A detailed description for each of these previously mentioned behaviors is provided in Figure 2. We opted to implement these categories as some studies used distinctive terms to refer to the same behavior. For example, Bailoo et al. (2014) used the term “snout contact with pups,” or “rapid movement around the pups” when referring to the “contact with pups” category. Regarding harmful/adverse caregiving behavior, some studies use several terms to refer to this behavior, such as “step on,” “drop,” “drag,” “avoid,” and “roughly handle” (Blaze et al., 2013; Asok et al., 2014; Doherty et al., 2016). Using all the different terms included in the studies would not be a feasible way of rendering a comparison between the results, which makes our categorization essential for the execution of this review.

## Results

The database search yielded 1,107 studies. After removing duplicate records (*n* = 410), a total of 697 papers were reviewed. Following application of exclusion criteria, 56 papers were included in this systematic review.

A total of 12 different early life stress protocols were identified in the reviewed studies:

- Early handling (Raineki et al., 2014), which briefly separates pups from the mother and exposes them to a new environment for up to 15 min;
- Maternal separation (Tractenberg et al., 2016), which separates pups are separated from the dam and exposed to a new environment; 15 min to 8 h);
- Maternal deprivation (Llorente-Berzal et al., 2012) (pups are separated from the dam and exposed to a new environment for a long period; 8–24 h);
- Limited bedding (Rice et al., 2008) (the dam and the pups are exposed to an impoverished environment during early development);
- Sibling deprivation (Li et al., 2008) (only one pup remains with the dam, while the rest of the offspring is sacrificed);
- Sibling separation (De Almeida Magalhães et al., 2018) (half of the litter remains with the dam, while the other half is separated and exposed to a new environment);
- Nutritional deprivation (Crnic, 1980) (pups are left for a period with a dam with cauterized teats, which precludes nursing);
- Maternal maltreatment (Blaze and Roth, 2017) (litter is exposed to a stressed dam outside the home cage);
- Repeated cross-fostering (Luchetti et al., 2015) (pups change caregiver during early development);
- Male intruder (Cirulli et al., 2007) (the dam and pups are kept in their home cage with an unfamiliar male for a brief time);
- Unpredictable maternal separation with unpredictable maternal stress (Franklin et al., 2010) (pups are separated from the dam and exposed to a new environment, and the dams are exposed either to restraint stress or forced swim stress);
- Adoptive mother (Fuentes et al., 2014) (the pups are exposed to a substitute mother and a limited amount of bedding for a short period).

### Characteristics and Summary of Studies

Regarding the included studies, 69.6% (*n* = 39) of them were performed with rats and 30.4% (*n* = 17) were performed with mice. When analyzing data from rat studies, we observed that Sprague-Dawley (30.95%; *n* = 13) was the primary strain used to evaluate maternal behavior. Wistar (28.57%; *n* = 12) and Long-Evans (26.19%; *n* = 11) were also commonly used strains. All other strains in this review represented only 11.91% (*n* = 6) of the studies, while one study did not report the utilized strain (2.38%). In 79.48% of the studies, researchers controlled the litter size (*n* = 31), which ranged from 6 to 14 pups, with an average of 9.8 pups per litter. In relation to the early life stress protocols utilized, the most common protocols were maternal separation (38%, average of 155 min; *n* = 19), followed by limited bedding (22%, average of 8 days; *n* = 11), early handling (18%, average of 16 min; *n* = 9) and maternal maltreatment (10%, average of 30 min; *n* = 5). The sum of all other protocols used accounted for only 12% (*n* = 4). Regarding the evaluation of maternal behavior, the maximum number of evaluation days was 21, and the minimum was 1. On average, there were 9 days of maternal care assessment. The daily observation of maternal behavior ranged from 30 to 480 min, with an average of 175 min of observation per day. The average time between each observation was 4.9 min.

In terms of the studies using mice, we determined that the majority of studies used C57BL/6 (66.7%; *n* = 12). Other strains, such as BALB/c (11.1%; *n* = 2), NMRI (11.1%; *n* = 2), and CD1 (11.1%; *n* = 2) were less represented. In 58.8% of studies, the researchers controlled the litter size (*n* = 10), and the average number of pups per litter was 6.8. Maternal separation (40%, average of 176 min; *n* = 8) was the most used early life stress protocol, followed by limited bedding (25%, 8 days; *n* = 5) and early handling (10%, 15 min; *n* = 2). The sum of all other early life interventions accounted for 25% (*n* = 5). The average duration of maternal behavior evaluation for mice studies was 8.6 days, while daily observation ranged from 30 to 480 min (average of 116 min). The average time between each maternal observation was 2.26 min. The descriptive characteristics and summary of the included studies are presented in Supplementary Table 1 for rat data and Supplementary Table 2 for mice data.

### Methodological Quality Assessment

Among all studies analyzed, the maximum score obtained was 13.5, the minimum was 8.5; the average across the studies was 11.5. The methodological quality of each study is presented in the last column of Supplementary Table 1 for rat studies and Supplementary Table 2 for mice studies.

It may be worth noting that some important methodological aspects observed in mice and rat studies are described in 100% of the papers, such as the following: light conditions, experimental groups, age beginning/final, ELS duration and time, MB time and outcomes. Moreover, in mice studies, strain, cage description, animals per group, CT description, ELS description, and MB duration were also reported in 100% of the studies. Only one rat study did not mention the strain used in the experiments. Overall, few studies gave information about any samples lost (animals that were excluded for any reason): 30.77% for rats and 11.76% for mice. Moreover, only 12.82 and 5.88% in rats and mice, respectively, used blinding methods to collect maternal behavior. Only 25.64% of the studies with rats gave information about breeding procedures, compared to 64.71% in mice. The remaining items on the checklist were reported in at least 50% of the studies. Finally, the average percentage of all items for rat and mice studies was similar (80.85 and 83.61%, respectively) (details in Supplementary Figure 2 for rat studies and Supplementary Figure 3 for mice studies).

### Overall Early Life Stress Impact on Maternal Behavior

**Figure 3:**
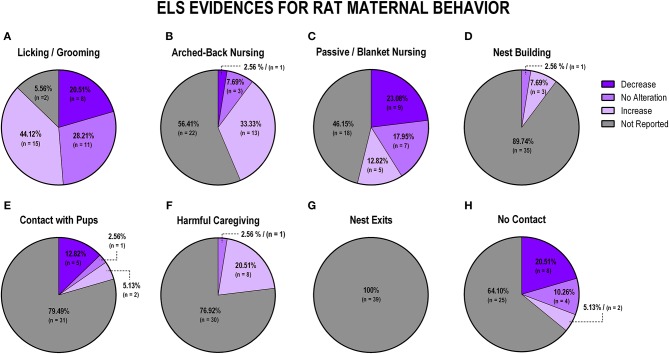
Percentage of behavior findings from all rat studies. Licking and grooming behavior **(A)**. Arched-back nursing behavior **(B)**. Passive and blanket nursing behavior **(C)**. Nest building behavior **(D)**. Contact with pups behavior **(E)**. Harmful caregiving behavior **(F)**. Nest exits behavior **(G)**. No contact behavior **(H)**.

**Figure 4:**
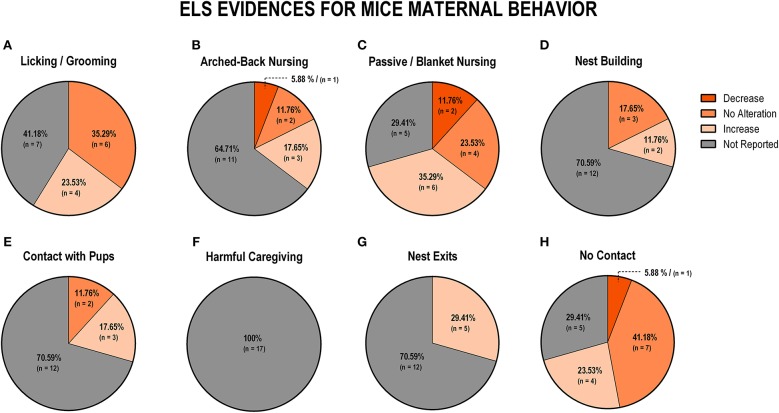
Percentage of behavior findings from all mice studies. Licking and grooming behavior **(A)**. Arched-back nursing behavior **(B)**. Passive and blanket nursing behavior **(C)**. Nest building behavior **(D)**. Contact with pups behavior **(E)**. Harmful caregiving behavior **(F)**; Nest exits behavior **(G)**. No contact behavior **(H)**.

To evaluate the overall impact of early life stress exposure on dam-pup interaction, we first grouped all the stress protocols and analyzed the alterations of the maternal behaviors included in this review. Figure 3 displays findings from rat studies, and Figure 4 shows findings from mice studies.

When analyzing the studies with rats that evaluated licking and grooming, we observed that most evidence indicates an increase of those behaviors (*n* = 15; 38.5%), although no alteration and decrease were also observed in some studies (*n* = 11; 28.2%; *n* = 8; 20.5%, respectively), while few studies failed to report this behavior (*n* = 5; 12.8%; Figure 3A). Arched-back nursing, which is a major component of maternal care, was not reported in the majority of the studies (*n* = 22; 56.4%), and when it was reported, it was mainly related to its increase after early life stress exposure (*n* = 13; 33.3%; Figure 3B). Passive and blanket nursing, which are less active nursing postures, were not reported in 18 studies (46.2%), but, when reported, they did not present in the same pattern as arched-back nursing, as most of the studies found a decrease or no alteration on those behaviors (*n* = 9; 23%; *n* = 7; 18%, respectively; Figure 3C).

Nest building, which is also essential for the pups' thermal regulation and development, was reported in only four studies, and most of these found an increase (*n* = 3; 7.7%; Figure 3D). The majority of the studies did not report dam-pup contact (*n* = 31; 79.5%), which is considered a maternal behavior with no active interaction between the dam and the pups, but 5 (12.8%) of the eight studies that reported this behavior found a decrease, when comparing stressed to control dams (Figure 3E). Regarding non-maternal behaviors, harmful caregiving was mostly discussed in terms of an increase (*n* = 8; 20.5%), but 76.92% (30 studies) did not evaluate this behavior (Figure 3F). The number of nest exits was not reported in any of the studies (*n* = 39; 100%; Figure 3G). Finally, off-nest behaviors (no contact), when reported (*n* = 25; 64.10% not reported) were mostly described in terms of a decrease (*n* = 8; 20.5%; Figure 3H).

Regarding mice data, the behaviors of licking and grooming were associated with no alteration (*n* = 6; 35.5%) or an increase (*n* = 4; 23.4%), while the remaining studies did not report those behaviors (*n* = 7; 41.2%; Figure 4A). Arched-back nursing was not reported in 11 studies (64.7%), but of the remaining studies, three found an increase (17.6%), two did not observe any difference (11.8%), and one study (5.9%) reported a decrease in this behavior (Figure 4B). An increase in a less effective nursing pattern, such as passive or blanket nursing was reported by six studies (35.3%), and no alteration in this behavior was reported by four studies (23.5%) (Figure 4C). The majority of the studies (*n* = 12; 70.6%) did not report nest building behavior, but no alteration was identified in three studies (17.6%), while two studies (11.8%) reported an increase in this behavior (Figure 4D). Maintaining contact, without actively interacting with the pups, was also not reported in 12 studies (70.6%), but three studies (17.6%) indicated an increase and two studies (11.8%) observed no difference between control and stressed groups (Figure 4E). When it comes to non-maternal behaviors, none of the 17 (100%) mice studies reported harmful caregiving (Figure 3F). All studies that reported the number of exits from the nest identified an increase (*n* = 5; 29.4%), but 12 studies (70.6%) did not report this behavior (Figure 4G). Finally, off-nest behaviors (no contact) were mostly related to reports of no alteration (*n* = 7; 41.2%), or an increase (*n* = 4; 23.5%), while five studies (29.4%) did not report this behavior (Figure 4H).

### Maternal Separation and Limited Bedding Studies

**Figure 5:**
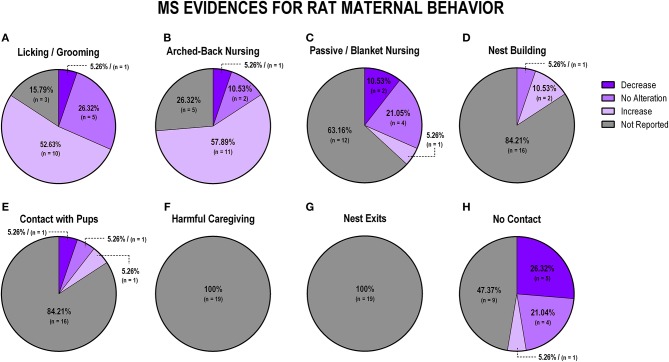
Percentage of behavior findings from MS rat studies. Licking and grooming behavior **(A)**. Arched-back nursing behavior **(B)**. Passive and blanket nursing behavior **(C)**. Nest building behavior **(D)**. Contact with pups behavior **(E)**. Harmful caregiving behavior **(F)**. Nest exits behavior **(G)**. No contact behavior **(H)**.

The majority of protocols utilized by the studies included in this review are maternal separation (MS) and limited bedding (LB). For this reason, we performed a secondary analysis to understand the specific impact of those protocols on maternal behavior. The use of MS and LB in rat and mouse studies accounted for 61.4% (43 protocols) of the 70 early life stress protocols included in this review. We identified 19 studies (38%) using MS and 11 (22%) using LB for rat studies, while MS was observed in 8 (40%) and LB in 5 (25%) protocols for studies using mice. The discrepancy between the number of studies included in the review (*n* = 56) and the number of protocols (*n* = 70) is due to the utilization, by some studies, of more than one early life stress protocol. Figure 4 displays findings from MS studies, and Figure 5 shows findings from LB studies.

### Maternal Separation Impact on Maternal Behavior

When evaluating the impact of MS exposure in rat studies (*n* = 19), we observed that the majority of the studies (*n* = 10; 52.6%) reported an increase in licking and grooming behaviors (Figure 5A). Following the same pattern, arched-back nursing was also reported to be majorly increased during MS exposure (*n* = 11; 57.9%; Figure 5B). When evaluating passive/blanket nursing, most studies did not report this behavior (*n* = 12; 63.2%), but the majority of the studies that did report it indicated no alteration (*n* = 4; 21%; Figure 5C). Nest building, when reported, was associated with an increase (*n* = 2; 10.5%) for MS protocols (Figure 5D). We identified heterogeneous results regarding maternal contact with pups, since one study (5.3%) showed an increase, one study (5.3%) showed no alteration, and one study (5.3%) showed a decrease in this behavior, but 16 studies (84.1%) did not report this behavior at all (Figure 5E). Harmful caregiving and the number of exits from the nest were not reported in any of the studies (Figures 5F,G, respectively). Off-nest behavior (no contact), when reported, was mostly related to a decrease (*n* = 5; 26.3%) or no alteration (*n* = 4; 21%; Figure 5H).

**Figure 6:**
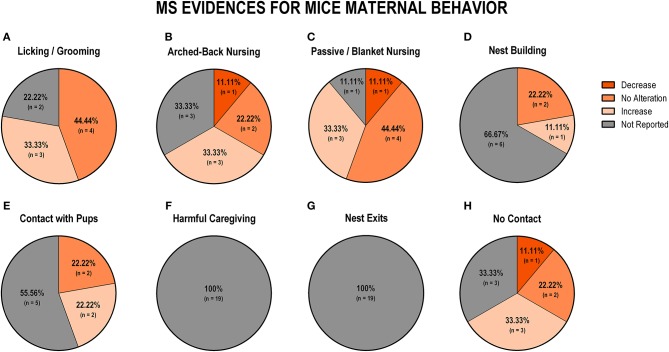
Percentage of behavior findings from MS mice studies. Licking and grooming behavior **(A)**. Arched-back nursing behavior **(B)**. Passive and blanket nursing behavior **(C)**. Nest building behavior **(D)**. Contact with pups behavior **(E)**; Harmful caregiving behavior **(F)**. Nest exits behavior **(G)**. No contact behavior **(H)**.

Regarding the evidence for the impact of MS in mice studies (*n* = 9), we observed that most studies showed no alteration of licking/grooming behavior (*n* = 4; 44.5%), while three studies reported an increase in these behaviors (*n* = 3; 33.3%; Figure 6A). Regarding arched-back nursing, three studies (33.3%) reported an increase, and another three studies (33.3%) did not report this behavior (Figure 6B). Regarding passive/blanket nursing, four studies (44.5%) showed no alteration, while three studies (33.3%) reported an increase in these behaviors (Figure 6C). Nest building was not reported by the majority of the studies (*n* = 6; 66.7%), and two studies (22.2%) showed no alteration in this behavior (Figure 6D). Regarding dam-pup contact, two studies (22.2%) showed an increase and two studies (22.2%) reported no alteration, while the remaining studies (*n* = 5; 55.6%) did not report this behavior (Figure 6E). None of the studies reported harmful behavior or the number of nest exits (Figures 6F,G, respectively). Finally, for off-nest behaviors (no contact) that were reported, three studies (33.3%) showed an increase, two studies (22.3%) reported no alteration, and one (11.1%) showed a decrease in this behavior (Figure 6H).

### Limited Bedding Impact on Maternal Behavior

**Figure 7:**
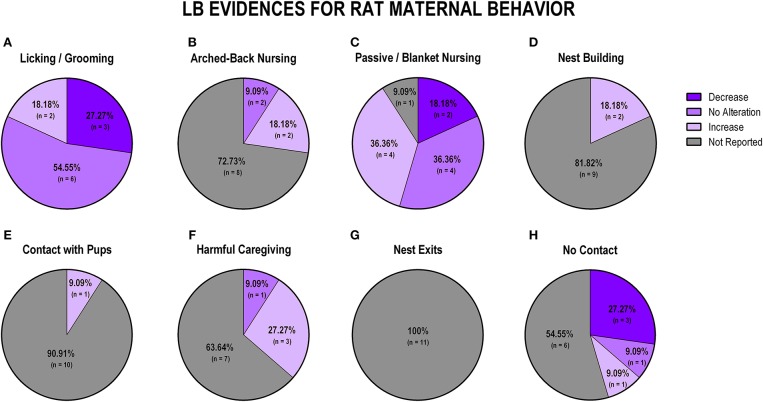
Percentage of behavior findings from LB rat studies. Licking and grooming behavior **(A)**. Arched-back nursing behavior **(B)**. Passive and blanket nursing behavior **(C)**. Nest building behavior **(D)**. Contact with pups behavior **(E)**. Harmful caregiving behavior **(F)**. Nest exits behavior **(G)**. No contact behavior **(H)**.

When analyzing data from LB studies in rats (*n* = 11), we identified that most of the studies showed no alteration of licking/grooming behavior (*n* = 6; 54.5%; Figure 7A). Further, 72.2% (*n* = 8) of the studies did not report arched-back nursing behavior, while two studies (18.2%) indicated an increase and one study (9.1%) showed no alteration in this behavior (Figure 7B). Regarding passive/blanket nursing, no alteration (36.35%) and increase (36.35%) were both reported in four studies, respectively (Figure 7C). Only 2 (18.2%) studies reported nest building behavior, and both of them identified an increase in this behavior (Figure 7D). Contact with pups was only evaluated in one study, which reported an increase in this behavior (*n* = 1; 9.1%; Figure 7E). Harmful caregiving was mostly related to an increase (*n* = 3; 27.3%; Figure 7F). None of the 11 studies reported the number of nest exits (Figure 7G). Most of the studies that reported off-nest behaviors (no contact) reported a decrease (*n* = 3; 27.3%; Figure 7H).

**Figure 8:**
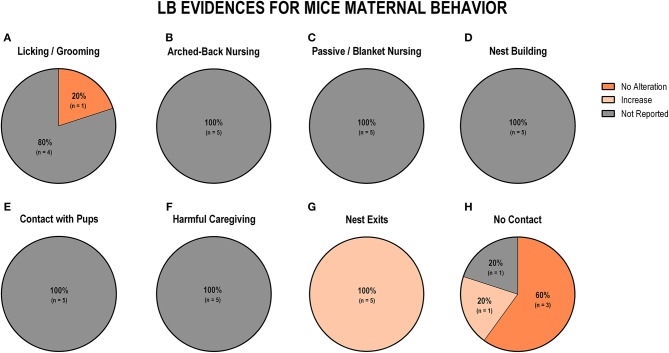
Percentage of behavior findings from LB mice studies. Licking and grooming behavior **(A)**. Arched-back nursing behavior **(B)**. Passive and blanket nursing behavior **(C)**. Nest building behavior **(D)**. Contact with pups behavior **(E)**. Harmful caregiving behavior **(F)**. Nest exits behavior **(G)**. No contact behavior **(H)**.

Regarding the evidence for LB in mice (*n* = 5), we noted that the only study that reported licking/grooming behavior did not observe differences between groups (*n* = 1; 20%; Figure 8A). Arched-back nursing, passive/blanket nursing, nest building, and contact with pups were behaviors that none of the studies reported (Figures 8B–E). Regarding non-maternal behaviors, harmful caregiving was also not reported by any of the studies (Figure 8F). All five studies (100%) reported an increase in the number of exits from the nest (Figure 8G). Three studies (60%) reported no alteration in off-nest behaviors (no contact), while one study (20%) indicated an increase, and one study (20%) did not report this behavior (Figure 8H).

## Discussion

Maternal behavior is a complex feature of rearing conditions that includes a variety of behaviors such as nursing (arched-back, passive), nest building, licking, and grooming the pups. Observations often include non-maternal behaviors such as eating, drinking, self-grooming, or no contact with pups. It was proposed that each component of maternal behavior may reflect unique neural circuitry, but it would be biologically useful for the circuitries to overlap to regulate a group of behaviors that appear together (Gammie, 2005). In this sense, two of the most common pup-oriented maternal behaviors that occur after birth are licking/grooming the pups and arched-back nursing (ABN). Licking/grooming (LG) the body as well as anogenital regions are an important stimulation that has already been shown to play a key role in pup neurodevelopment (Champagne et al., 2003, 2007). This behavior usually precedes the initiation of a nursing bout. It has already been shown that naturally occurring variations in the frequency of licking and grooming may predict neurobiological and behavioral outcomes (Champagne, 2008). To address that, early studies observed the maternal behavior over the first 8 days after birth in normally reared animals. Liu et al. (2000) demonstrated that pups raised by low-LG/ABN dams showed impaired spatial learning in the Morris water maze and hippocampal synaptogenesis, relative to pups raised by high-LG/ABN dams. When adults, these animals also showed reduced levels of the plasma adrenocorticotropic hormone, corticosterone levels' responses to acute stress and increased hippocampal glucocorticoid receptor expression (Liu et al., 1997). In fact, increased levels of LG and ABN in the first weeks of life plays a major role in the developmental processes involved in behavioral, endocrine and cognitive responses to stress later in life (Jensen Peña and Champagne, 2013).

One question that remains unanswered is whether early life stress alters the maternal care pattern or merely decreases the amount of time spent with the pups. It has already been shown that stress decreases the frequency of maternal LG and ABN in mothers who were previously identified as High-LG/ABN and then exposed to prenatal stress, suggesting that insults during the gestational period were able to completely reverse the maternal care pattern (Champagne and Meaney, 2006). Thus, it is reasonable to hypothesize that early life stress may change the maternal care pattern over the post-natal period. For example, Own and Patel (2013) showed that 15 min-MS and 180 min-MS exposure increased maternal care, specifically after reuniting the pups with the dams. Moreover, Pryce et al. (2001) showed that early handling also yielded an increase in arched-back nursing across several time points and that 4 h of maternal separation increased licking and arched-back nursing at the time of dam-litter reunion. Biggio et al. (2014) showed that the total frequency of ABN and LG were increased in dams exposed to maternal separation across postpartum days 3–15. However, this increase was specific to the time-point that coincides with the reunion period (post-MS). The same increased licking and arched-back nursing was observed by Couto-Pereira et al. (2016) also singularly in the observation period after MS procedures. These previously cited works makes it reasonable to suggest that the MS protocol exerts an effect that engages dams in more active postures of maternal care toward the pups, such as licking/grooming and arched-back nursing. This effect is not consistent across the included studies of this review. Bedrosian et al. (2018) suggested that MS-exposed dams were more likely to be highly maternal than control dams, although no statistically significant difference was observed. Der-Avakian and Markou (2010) found a decrease in licking/grooming prior to MS during the first and second postnatal week, together with a decreased arched-back nursing frequency, specifically in the second postnatal week. Moreover, Romeo et al. (2003) observed similar frequencies in behaviors such as arched-back nursing, licking and grooming, as well as off-nest and nest building behaviors. Such marginal differences may be explained by the timing of observations, taking into account the previously mentioned effect of increased maternal care following maternal separation. Using a modified version of the classical maternal separation protocol, Gracia-Rubio et al. (2016) separated the pups from their dams for 4 h per day from P2 to P5 and then increased to 8 h daily from P6 to P16. Pups were weaned early on P16 and they called this model maternal separation with early weaning (MSEW). They found increased arched-back nursing from P3 to P8 and that MSEW dams spent more time in the nest when compared to control dams. This data set should be interpreted with particular caution, as several studies did not report important components of maternal care. For rats, only two studies did not report licking/grooming observations but, regarding ABN observations, this number jumps to 22 (more than half). In mice, the number of studies that failed to report licking/grooming and arched-back nursing was also high (7 and 11, respectively). In fact, our review leads us to suggest that maternal separation, regardless of variation in period and duration, promotes an overall increase in maternal behavior after reunion with pups. It is believed that this increase in maternal care, following maternal separation, is a compensatory effect elicited by the off-nest period. Even though it appears that pups exposed to maternal separation experience extra maternal care, relative to control animals, this evidence shows that maternal separation alters the natural conditions whereby dams take care of their pups, which is an essential aspect of the long-lasting alterations caused by this model. We suggest that future studies using maternal separation models should always report both pre- and post-MS maternal behaviors in an attempt to control for this effect.

Due to the fact that alterations of the time of MS exposure may impact the maternal behavior differently, we sought to investigate the impact of BMS and PMS on licking/grooming and arched-back nursing (Supplementary Figure 4). Both prolonged and brief MS studies with rats yielded similar results, an increase in licking/grooming and arched-back nursing in the animals exposed to stress, relative to controls. When evaluating studies with mice, we observed that studies with prolonged protocols of MS showed no alteration in licking/grooming and heterogeneous results regarding arched-back nursing. Brief protocols of MS presented an increase in licking/grooming and arched-back nursing, but it is important to observe that only three studies that evaluated licking/grooming and one study that analyzed arched-back nursing with brief MS protocols. Overall, it is clear that both BMS and PMS increase maternal care in rats, but that this claim cannot be conclusive, due to a lack of evidence in studies with mice.

More recently, a novel model to disrupt maternal care was developed and has received growing attention from several laboratories around the world. The limited bedding model, characterized by limiting the amount of bedding material available for the dam and, therefore, creating a stressful environment for the dam that demands alteration of the pattern and quality of maternal care. Indeed, it has already been shown that this limited bedding model increased corticosterone levels in the dam on PND9 (Ivy et al., 2008). When this model was initially developed, authors reasoned that it is not only time spent with pups that is crucial, but also the quality of maternal care provided by dams when they are actually with pups in the rearing environment. To construct the limited bedding protocol, two methods limiting access to bedding materials have been used in the past two decades: (1) inserting a wire mesh floor in the cage to prevent dams from retrieving the bedding and incorporating it into their nests (Gilles et al., 1996); and (2) providing a very small amount of bedding and nesting material without the wire mesh (Roth and Sullivan, 2005). This model, with very few manipulation requirements, has sufficient flexibility to be performed in different periods and for various durations. This flexibility is a double-edged sword, given that specific periods and timing of development could be investigated, but this will also increase procedural variability across laboratories, making it difficult to compare works done in different laboratories.

To date, studies that looked at maternal care following limited bedding exposure reported mixed results. Most studies showed that limited bedding exposure did not change the frequency of licking and grooming in rats, although two studies from the same laboratory reported that limited bedding increased frequency of licking and grooming from PND2 to PND7 (Fuentes et al., 2014, 2018). It is important to note that the authors assembled both licking and nursing data within the same scores, making it difficult to separate out each component for comparison. On the other hand, a reduction in the time spent nursing or licking and grooming the pups by dams exposed to limited bedding, relative to controls was observed in two studies (Ivy et al., 2008; Moriceau et al., 2009). In mice, although only one study reported licking and grooming frequency, with no differences between LB dams and controls, several others studies did not report this important component of maternal care after exposure to limited bedding. However, limited bedding protocols are thought to have a different impact on the maternal care pattern, relative to the maternal separation protocol. Rather than just decreasing the amount of time available to nurture the pups, as is the case with maternal separation, limited bedding promotes more fragmented and sometimes erratic nurturing behaviors (Walker et al., 2017). Even though harmful caregiving is considered to be a significant component of the limited bedding model, we observed that the majority of the studies did not investigate this behavior. Surprisingly, an increase in this behavior was only observed in three studies. We determined that, due to the difficulties of analyzing harmful caregiving during natural cage conditions, studies must perform specialized tests to evaluate this behavior. Nevertheless, those studies were not included in this review because one of the inclusion criteria was investigation of the natural occurrence of each behavior. All of the limited bedding studies of mice included in this review showed that dams exposed to limited bedding show an increase in the number of nest exits. This specific aspect of the limited bedding manipulation produces important changes in the pattern of maternal care, resulting in shortened bouts of each nurturing behavior.

Over the course of this review, we recognized that investigating the overall impact of several early-life stress protocols on maternal behavior does not elucidate the general question of how the stressor protocols impact maternal care behavior. The main reason is that each model has specific methodological features that promote distinct patterns of maternal behavior. To demonstrate this variability, we analyzed the standard deviation of the overall ELS impact on maternal behavior (Supplementary Figure 5). It is clear that essential maternal behaviors, such as, licking/grooming, nursing, and the overall contact with pups are highly variable, especially in studies using rats. This variability exemplifies how difficult is to identify an overall construct without investigating the specific characteristics of each ELS model. Moreover, looking at the ELS studies together may dilute specific effects for each model. For this reason, we opted to undertake a deeper exploration of two of the most used models (MS and LB). A good example of a model with a specific maternal behavior pattern is the maternal maltreatment protocol, in which all studies pointed to an increase in maternal aggression. Furthermore, several models included only one study that evaluated maternal behavior, which renders a more in-depth analysis of maternal behavior patterns impossible. In addition to the models that have minimal evidence, some maternal behaviors are frequently neglected. Nest building, contact with pups, pup retrieval (naturally, not the test), time off-nest and harmful behavior were also poorly reported among the included studies, making it difficult to compare the studies. For example, a major component of maternal care, which is nest building, was only reported in 10.25% of rat studies included in this review.

### Methodological Considerations

The assessment of variations in maternal behavior in laboratory mice and rats is an onerous and time-consuming method. This fact may explain why our review found that studies range from 2 to 21 days of observation, and from 30 to 480 min per day. Sometimes, when maternal behavior is not a major outcome of the study, researchers tend to shrink observations periods on a few days after birth. In animal studies, the manipulation of dam-pup interaction is the one of the basic premises of early life stress models. However, several studies that used an early-life stress protocol generally do not report aspects related to maternal care. In this review, we showed that studies using early-life stress should observe and report variations in maternal care following ELS exposure, considering that the specific elicited by every ELS elicits may have the capacity to change maternal behavior of the dam toward the pups. Another possibility is to use tests of maternal care in dams, focused on behaviors, such as maternal aggression toward an intruder or pup-retrieval motivation. Although these tests may provide insightful measures of maternal care, they do not reproduce the environment of natural rearing conditions. Our review did not include results of maternal behavior tests, only home-cage observations, as we were interested in the impact of ELS models on maternal behavior in home-cage conditions, with the ELS model being the only major environmental manipulation.

It is important to highlight that the maternal separation procedures are highly variable in terms of several key factors, such as separation period, separation duration, temperature regulation and pup isolation. The applied procedures in MS procedures made the comparison between studies far too complex. Schmidt et al. (2011) describe inconsistency in the literature regarding a depression-like phenotype elicited by maternal separation studies, mostly due to uncontrolled factors that had the ability to influence the outcomes. In addition, Nylander and Roman (2013) reported that there is no unique model of maternal separation in rodents but rather, several models, with several different effects and outcomes. Despite all the criticism regarding separation models, these models provided invaluable insights into the neurobiology of early-life stress over time and are integral to the study of the impact of early-life stress on drug seeking behavior (Huot et al., 2001; Cruz et al., 2008; García-Gutiérrez et al., 2016), anxiety-like behaviors (Romeo et al., 2003) and depression-like behaviors (Schmidt et al., 2011; Vetulani, 2013; Andersen, 2015). Our group recently conducted a systematic review on animal studies to explore the MS effects on behavioral outcomes and found that maternal separation studies do not follow the guidelines for reporting animal research and that several inconsistencies in MS procedures may influence behavioral outcomes (Tractenberg et al., 2016). Nevertheless, to push the field even further, it is critical to adopt standard procedures, across all laboratories, regarding MS, and to ensure that outcomes and observations are accurately reported in published works.

Regarding the methodological quality of the included studies, there are factors that may influence not only maternal care, but other outcomes of the study as well. For example, only 25.64% of the studies with rats reported breeding procedures, which, for some studies, may not have an impact on the research outcome, but when discussing maternal care, the breeding protocol is an essential piece of information. When pregnant females are bought directly from animal facilities, and not bred in-house, the dams are exposed to several days of gestational stress during transportation and carrying that are not always performed with due precaution. Our study demonstrated that, in 43.6% of studies with rats, researchers buy pregnant dams from animal facilities, while for mice studies 76.5% are bred in-house, reducing gestational stress. This is alarming for researches working with rats, as several studies have already shown that maternal stress can promote altered maternal care and behavioral outcomes (Patin et al., 2002; Weinstock, 2017). Furthermore, differences in how the control group was bred and treated (animal facility reared, handled or with environmental enrichment) may influence the maternal care comparison. In our review almost all studies used animal facility reared controls (92.3% for rats and 100% for mice), which is when the animals are only manipulated during regular cage cleaning and eventually to weigh the pups. For studies that aim to evaluate the impact of early life stress on maternal behavior, we suggest using animal facility reared controls. This is partly because doing so will facilitate comparisons in the context of the studies, but also because handling procedures or providing environmental enrichment during early development are well-documented as a possible way of altering the regular neurodevelopment processes of rodents (Wilson et al., 1986; Hullinger et al., 2015). Interestingly, none of the evaluated studies achieved the maximum methodological score. One reason for that may be that most of the time, each laboratory utilizes its own methodology, which results in difficulties standardizing the procedures, thereby lowering the quality score. Moreover, there are times that the methodology used in a given study is not fully included in the final manuscript, which interferes with the study's replicability and lowers the overall methodological score. We suggest that the studies utilize a methodological checklist to improve the description of the experiments, and to facilitate and enhance the replicability for further studies (Kilkenny et al., 2010; Hooijmans et al., 2011).

Even though our review is focused on the impact of ELS on maternal behavior, we sought to report on the mode of conducting the follow-up of the pups. Few studies failed to perform any pup follow-up (15.4% for rats and 5.9% for mice). Some studies performed only behavioral or biological measures (23.1% for rats and 23.6% for mice). Nevertheless, the vast majority of studies performed the follow-up by investigating behavioral and biological factors (61.5% for rats and 70.5% mice), which is critical to determine how maternal care during early development can influence several parameters throughout long-term development. Furthermore, based on the NIH directive concerning the use of females in biomedical studies (NOT-OD-15-102, NIH, 2015), we determined that 46.2% of rat studies and 29.4% of mice studies performed sex difference comparison, while 38.4% of the rat studies and 64.7% of the mice studies used only male animals or did not report male x female comparison. Taking into account several pieces of evidence, which showed that dams tend to spend more time actively nourishing male pups (Moore and Morelli, 1979; Richmond and Sachs, 1984; Hao et al., 2011), we suggest that the follow-up should be performed on both sexes, as the alterations in maternal care during early development may modulate the stress response of the pups (Chrousos and Gold, 1992).

### Study Limitations

We must this review's limitations, which are due mainly to the high variation of methodological approaches used to evaluate maternal behavior across studies. Each included study relied on a particular mode of assessment, differing in duration, times per day and which behaviors were selected for evaluation. These variable methodological characteristics, and the results obtained did not allow us to perform a meta-analysis. Also unclear are optimal duration of observation and which period of the day is most suitable to generate an accurate measure that better represents the maternal care pattern of a given dam. It is known, however, that maternal care consistency changes in response to light and dark cycles (Walker et al., 2017), so observations in different phases of the light cycle should be performed to facilitate better identification of maternal care. Some ELS protocols, such as sibling separation, maternal maltreatment, and strange male exposure, were applied only in a few studies, which prevented a more in-depth comparison of each ELS protocol. In addition, during the process of this review, the authors struggled to obtain clear and detailed information regarding the methodological procedures adopted in some studies. For this reason, we cannot exclude the possibility of a subjective bias in our evaluation processes. To minimize this risk, all evaluations were made by two or three independent authors. In the event of any discordance during this process, the reviewers would have a discussion to reach a consensus; if this still proved insufficient to establish consensus, the senior author was consulted. Further, we focused only on mice and rat studies, and did not consider other species; we opted to limit our review to these two species because of their use in the majority of studies with animal models and to enable a better comparison. Finally, the present review only included articles published in English, which may have excluded findings and evidence reported in other languages.

## Conclusion

**Figure 9:**
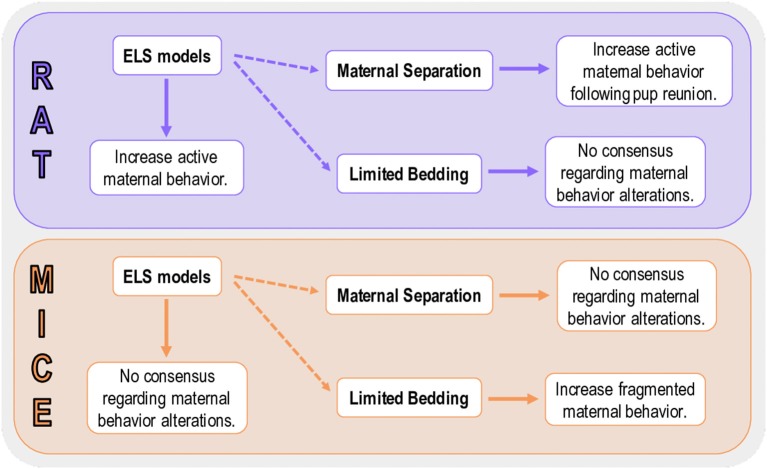
Summary of the main findings for rat and mice studies. Active maternal behavior compromises behaviors that are actively performed by the dam and promote a profound impact on pup development (e.g., licking/grooming and arched-back nursing). Fragmented maternal behavior is performed when the dam exits the nest several times, but the overall amount of maternal care is not necessarily altered.

Our data demonstrated that ELS models with different characteristics can promote specific maternal patterns of behavior, which can increase the difficulty of rendering a model-to-model comparison regarding the impact on maternal care. When discussing data from the most common protocol of this review (maternal separation), we suggest that, independently of the variations in MS protocols, an overall increase of the more important maternal behaviors, such as, nursing and licking/grooming is observed. This increase in maternal care may be an attempt to overcompensate for the time off-nest. Even though several studies used the LB model, it is very difficult to interpret the results in terms of maternal care, as most of the studies fail to report most maternal behaviors, due to methodological characteristics of the model itself. The only conclusive data, regarding LB, is that maternal behavior is fragmented during the protocol, but the amount of maternal care is not (Figure 9). Independent of the ELS model utilized, we suggest that methodological approaches to evaluate maternal behavior, such as time, duration, and the specific behavior selected for evaluation be more homogeneous across studies. Facilitating a better understanding of how ELS impacts maternal care studies requires consideration of all the behaviors included in this systematic review.

## Data Availability

No datasets were generated or analyzed for this study.

## Author Contributions

RO, KC, and ST conceived the study idea. RO and KC screened the abstracts and full texts. RO, LW-S, and RG-O performed data-analysis. RO, KC, LW-S, and TW wrote the manuscript. RO, KC, LW-S, TW, ST, FB, and RG-O critically revised the manuscript. RO, KC, LW-S, TW, ST, FB, and RG-O had access to all the data included in this systematic review and approved the submitted version.

### Conflict of Interest Statement

The authors declare that the research was conducted in the absence of any commercial or financial relationships that could be construed as a potential conflict of interest. The reviewer HB declared a past co-authorship with one of the authors RG-O to the handling Editor.
