# Supplementary material for: How Early Life Stress Impact Maternal Care: A Systematic Review of Rodent Studies
Source: Front Behav Neurosci. 2019 Aug 28;13:197. doi: 10.3389/fnbeh.2019.00197 (PMC6724664; doi:10.3389/fnbeh.2019.00197)
Supplement: Supplementary file 1 [file Table_1.DOCX]

**Table S1**. Descriptive characteristics, summary and main findings of rat studies.

| **Author (year)** | **Strain** | **Litter Size Control** | **Cross Fostering** | **Stress Protocol** | **Stress Period** | **Stress Duration** | **PND of behavior** | **Time of behavior observation** | **Findings** | **Quality Score (out of 14)** |
| --- | --- | --- | --- | --- | --- | --- | --- | --- | --- | --- |
| Alteba (2016) | NR | No | NR | Limited Bedding | PND 7-14 | 8 days | NR | 15 min; 3x/day | Increased stepping or jumping on, rough handling, isolating and tossing around; decreased nursing, licking and gathering; no significant differences in feeding, cleaning itself and exploring. | 9,5 |
| Asok (2014) | Long-Evans | Yes (12 pups) | NR | Maternal Maltreatment | PND 1-7 | 30 min | PND 1-7 | 30 min (observed every 5 min); 1x/day | Increased stepping on, dropping, dragging, avoiding and roughly handling; decreased licking, grooming, crouching over and nursing. | 12,5 |
| Benetti (2007) | Wistar | Yes (8 pups) | NR | Novelty Exposure | PND 1-10 | 15 min | PND 1, 5 and 10 | 30 min; 1x/day | No significant differences in licking. | 10 |
| Biggio (2014) | Sprague-Dawley | Yes (10-12 pups) | NR | Maternal Separation | PND 3-15 | 3 hours | PND 3-15 | 75 min; 4x/day | Increased arched-back nursing and licking. | 11,5 |
| Blaze (2013) | Long-Evans | NR | NR | Maternal Maltreatment | PND 1-7 | 30 min | PND 1-7 | 30 min (observed every 5 min); 1x/day | Increased stepping on, dropping, dragging, avoiding and roughly handling; decreased licking, grooming, crouching over and nursing. | 12 |
| Blaze (2015) | Long-Evans | Yes (10-12 pups) | NR | Maternal Maltreatment | PND 1-7 | 30 min | PND 1-7 | 30 min (observed every 5 min); 1x/day | Increased stepping on, dropping, dragging, avoiding and roughly handling; decreased licking, hovering and nursing. | 13 |
| Blaze (2017) | Long-Evans | Yes (10-12 pups) | NR | Maternal Maltreatment | PND 1-7 | 30 min | PND 1-7 | 30 min (observed every 5 min); 1x/day | Increased stepping on, dropping, dragging, avoiding and roughly handling; decreased nurturing behaviors. | 12 |
| Boccia (2006) | Long-Evans | Yes (10 pups) | Yes | Maternal Separation | PND 3-14 | 15 min or 3 hours | PND 2-6 | 240 min (observed every 4 min); 2x/day | No significant differences in licking. | 9,5 |
| Braw (2009) | Wistar, WKY, Sprague-Dawley and FSL | Yes (6 pups) | NR | Limited Bedding | PND 2-9 | 8 days | PND 4 and 9 | 60 min (observed every 10 min); 1x/day | Increased contact with the pups; increased nursing postures (strain WKY); decreased self-directed behaviors (strain WKY); no significant differences in licking, resting and motor activity. | 10,5 |
| Chocyk (2013) | Wistar | Yes (8 pups) | NR | Maternal Separation | PND 1-14 | 3 hours | PND 1-21 | 20 min (observed every 4 min); 8x/day | Increased arched-back nursing, total nursing and licking; increased hovering (after stress); decreased hovering (before separation, PND1-7); decreased time of nest; no significant differences in blanket and passive nursing. | 12,5 |
| Couto-Pereira (2016) | Wistar | Yes (6-8 pups) | NR | Maternal Separation / Handling | PND 1-10 | 3 hours / 10 min | PND 1-10 | 72 min (observed every 3 min); 5x/day | Increased arched-back nursing and licking; increased time off-nest (morning); decreased nursing (morning). | 11,5 |
| Crcnic (1980) | Sprague-Dawley | Yes (8 pups) | NR | Nutritional Deprivation | PND 2-21 | 2 hours / 12 hours | PND 1-21 | NR; 3x/day | Increased time in the nest (9hrs and 12hrs, PND1-10); increased time in the nest (9hrs and 16hrs, PND 11-20); no significant differences in eating. | 9,5 |
| Dalle Molle (2012) | Wistar | NR | NR | Limited Bedding | PND 2-9 | 8 days | PND 1-9 | 72 min (observed every 3 min); 5x/day | Increased nursing; decreased time off the nest; no significant differences in licking and grooming. | 12 |
| Daskalakis (2014) | Wistar | Yes (8-10 pups) | No | Maternal Separation / Handling | PND 3-5 | 8 hours / 15 min | PND 1-7 | 60 min (observed every 3 min); 5x/day | Increased arched-back nursing, licking and grooming (after stress). | 11,5 |
| De Azevedo (2010) | Wistar | Yes (8 pups) | NR | Maternal Separation / Handling / Tactile Stimulation | PND 1-10 | 10 min / 10 min/ 10 min | PND 1, 5 and 10 | 30 min; 1x/day | Increased licking (after stress); no significant differences in arched-back nursing, gathering the pups and time in the nest. | 10,5 |
| Der-Avakian (2010) | Long-Evans | No | NR | Maternal Separation | PND 1-14 | 3 hours and 30 min | PND 1-14 | 60 min (observed every 4 min); 1x/day | Decreased licking and grooming; decreased arched-back nursing (PND8-14). | 11 |
| Doherty (2016) | Long-Evans | Yes (10-12 pups) | NR | Maternal Maltreatment | PND 1-7 | 30 min | PND 1-7 | 30 min (observed every 5 min); 1x/day | Increased roughly handle, actively avoid, dropping and stepping on; decreased licking, grooming, nursing and hovering. | 9,5 |
| Fenoglio (2006) | Sprague-Dawley | No | Yes | Maternal Separation / Handling | PND 9 / PND 1-13 | 3 hours / 15 min | PND 2-8 | 36 min (observed every 3 min); 1x/day | Increased licking and grooming (after stress). | 10 |
| Francis (2008) | Long-Evans | Yes (6-7 pups) | NR | Maternal Separation | PND 2-14 | 15 min or 3 hours | PND 2-14 | 60 min (observed every 4 min); 2x/day | Increased licking and grooming (after stress). | 8,5 |
| Fuentes (2014) | Long-Evans | Yes (maximum 12 pups) | No | Limited Bedding / Substitute Mother | PND 1-7 | 7 days / 1 hour | PND 1-7, 13 and 18 | 75 min (observed every 3 min); 4x/day | Increased arched-back nursing; increased licking and grooming (at night); decreased off-nest behavior; no significant differences in blanket nursing and spine nursing. | 13 |
| Fuentes (2018) | Long-Evans | Yes (maximum 12 pups) | No | Limited Bedding | PND 2-9 | 8 days | PND 2-8, 13 and 18 | 75 min (observed every 3 min); 4x/day | Increased arched-back nursing, licking and grooming; decreased time off the nest; no significant differences in blanket nursing and spine nursing. | 13,5 |
| Guadagno (2017) | Sprague-Dawley | Yes (8-10 pups) | NR | Limited Bedding | PND 1-9 | 9 days | PND 5 and 6 | 72 min (observed every 1 min); 3x/day | Increased nursing; no significant differences in grooming and fragmented behavior. | 12 |
| Ivy (2008) | Sprague-Dawley | Yes (12 pups) | NR | Limited Bedding | PND 2-9 | 8 days | NR | 75 min (observed every 3 min); 3x/day | Increased epochs of pups out of nest; increased epochs away from the pups; decreased licking and grooming (dark-phase, PND2-5). | 11,5 |
| Kosten (2010) | Sprague-Dawley | Yes (12 pups) | NR | Maternal Separation / Handling | PND 2-9 | 1 hour / 5-10 min | PND 2-9 | 60 min (observed every 5 min); 1x/day | Increased time creating a huddle, licking and climbing; decreased time picking up pups to place outside the nest, self-grooming and burrowing. | 12,5 |
| Li (2008) | Sprague-Dawley | Yes | No | Sibling Deprivation | PND 1-21 / PND 7-21 | 14 days | PND 1-14 | 60 min; 2x/day | Increased licking and grooming (PND1-7). | 10,5 |
| Llorente-Berzal (2011) | Wistar | Yes (8 pups) | No | Maternal Deprivation | PND 9 | 24 hours | PND 6-8 and PND 10-12 | 75 min (observed every 3 min); 5x/day | Increased licking and grooming (PND10). | 13,5 |
| Macri (2004) | Lister-Hooded | No | NR | Maternal Separation / Handling | PND 1-13 / PND 2-8 | 4 hours / 15 min | PND 1-13 | 60 min (observed every 3 min); 8x/day | Increased active nursing; decreased self-maintenance. | 11 |
| Macri (2008) | Lister-Hooded | Yes (8 pups) | NR | Maternal Separation / Handling | PND 2-10 | 4 hours / 15 min (twice) | PND 2-8 | 60 min (observed every 6 min); 7x/day | Increased active nursing; increased maternal care (after stress). | 11,5 |
| Maniam (2010) | Sprague-Dawley | Yes (9-14 pups) | No | Maternal Separation | PND 2-14 | 15 min or 3 hours | PND 2-10 | 60 min (observed every 4 min); 8x/day | Increased arched-back nursing; decreased passive nursing and eating; decreased off-nest (light phase). | 11,5 |
| Mclaughlin (2016) | Sprague-Dawley | Yes (11-12 pups) | NR | Limited Bedding | PND 1-10 | 10 days | PND 2-9 | 72 min (observed every 3 min); 4x/day | Increased nursing (dark cycle); no significant differences in grooming and abusive or neglectful behavior. | 11,5 |
| Molet (2016) | Sprague-Dawley | Yes (12 pups) | Yes | Limited Bedding | PND 2-9 | 8 days | PND 2-9 | 50 min; 2x/day | No significant differences in nursing, arched-back nursing, licking and grooming. | 10 |
| Moriceau (2009) | Long-Evans | Yes (12 pups) | NR | Limited Bedding | PND 1-7 | 7 days | NR | 30 min; 2x/day | Increased nest building, stepping and rough handling of pups; decreased licking and nursing. | 10,5 |
| Moussaoui (2013) | Wistar | Yes (10-12 pups) | Yes | Limited Bedding / Maternal Separation | PND 2-10 / PND 2-9 | 9 days / 15 min | PND 2-9 | 50 min; 1x/day | Increased time building the nest, self-grooming, retrieving pups to the nest and number of pups off the nest; increased time moving (PND4); no significant differences in licking, grooming, eating, drinking and nursing in any type. | 11 |
| O’Malley (2011) | Sprague-Dawley | NR | NR | Maternal Separation | PND 2-12 | 3 hours | PND 2 | 30 min (observed for 1 min every 10 min); 3x/day | Increased arched-back nursing (after separation); no significant differences in grooming and pup grouping. | 10 |
| Ruedi-Bettschen (2006) | Fischer | Yes (8 pups) | NR | Maternal Separation / Handling | PND 1-14 | 4 hours / 15 min | PND 1-14 | 60 min (observed every 7.5 min); 3x/day | Increased licking (after stress); increased kyphosis (morning and after stress); decreased time off their pups (after stress). | 12 |
| Singh-Taylor (2018) | Sprague-Dawley | Yes (10 pups) | Yes | Handling | PND 2-9 | 15 min | PND 2-9 | 30 min; 1x/day | Increased licking and grooming (after stress). | 11,5 |
| Stamatakis (2015) | Wistar | No | No | Maternal Separation | PND 1-12 | 15 min | PND 1-12 | 30 min (observed every 5 min); 6x/day | Increased arched-back nursing, licking and grooming; decreased time off-nest (PND8-10). | 10,5 |
| Van der Dolen (2014) | Wistar | Yes | No | Maternal Separation | PND 2-14 | 3 hours | PND 2-8 | 60 min (observed every 3 min); 4x/day | Increased arched-back nursing (PND3-7); no significant differences in time away from the pups, contact with the pups, blanket-posture nursing, passive-posture nursing, licking and grooming. | 12 |
| Vilela (2017) | Wistar | Yes (8 pups) | NR | Maternal Separation | PND 2-15 | 3 hours | PND 2-15 | 90 min (observed every 3 min); 2x/day | No significant differences in licking, arched-back nursing, blanket posture nursing, passive nursing, nest building, feeding, exploring the cage housing, self-grooming. | 11,5 |

*Note:* Strain: WKY = Wistar-Kyoto; FSL = Flinders Sensitive Line. NR = Not Reported; PND = Postnatal Day.
