# Supplementary material for: How Early Life Stress Impact Maternal Care: A Systematic Review of Rodent Studies
Source: Front Behav Neurosci. 2019 Aug 28;13:197. doi: 10.3389/fnbeh.2019.00197 (PMC6724664; doi:10.3389/fnbeh.2019.00197)
Supplement: Supplementary file 2 [file Table_2.DOCX]

**Table S2**. Descriptive characteristics, summary and main findings of mice studies.

| **Author (year)** | **Strain** | **Litter Size Control** | **Cross Fostering** | **Stress Protocol** | **Stress Period** | **Stress Duration** | **PND of behavior** | **Time of behavior observation** | **Findings** | **Quality Score (out of 14)** |
| --- | --- | --- | --- | --- | --- | --- | --- | --- | --- | --- |
| Arp (2016) | C57BL/6J | No | NR | Limited Bedding | PND 2-9 | 8 days | NR | 45 min (observed every 3 min); 1x/day | Increased exits from the nest and number of pups out of the nest; no significant difference in time spent off-nest. | 12 |
| Bailoo (2014) | C57BL/6 | No | NR | Maternal Separation | PND 2-14 | 4 hours | PND 2-14 | 60 min (time in each activity); 2x/day | Increased time in the nest and licking; increased quiescent nursing (after stress); increased contact with the pups (PND 8, 10 and 12). | 12 |
| Bedrosian (2018) | C57BL/6 | NR | Yes | Maternal Separation | PND 1-14 | 3 hours | PND 1-14 | 40 min (observed every 4 min); 2x/day | No significant differences in arched-back nursing, licking/grooming and contact with pups. | 11 |
| Cirulli (2007) | CD1 | Yes (8 pups) | NR | Handling /Male intruder | PND 2-14 | 15 min / 5 min | PND 2, 4, 6, 8, 10, 12 and 14 | 80 min (observed every 5 min); 6x/day | Increased licking (PND 2); increased nursing (PND 10); no significant differences in nest building, resting alone and eating. | 11,5 |
| Franklin (2010) | C57BL/6J | No | No | Unpredictable Maternal Separation with Unpredictable Maternal Stress | PND 1-14 | 3 hours / 5 or 20 min | PND 1-14 | 30 min (observed every 1 min); 3x/day | Increased time off-nest (PND 1-7); decreased active nursing (PND 1-7). | 11 |
| Gracia-Rubio (2015) | CD1 | No | NR | Maternal Separation | PND 2-16 | 4 hours or 8 hours | PND 1-16 | 75 min (observed every 3 min); 3x/day | Increased arched-back nursing (PND 3-8); increased blanket nursing (PND 9-15); increased off-nest behavior (PND 3-14); no significant differences in licking, grooming and passive nursing. | 11 |
| Gunn (2013) | C57BL/6J | Yes (max 8 pups) | NR | Limited Bedding | PND 2-9 | 8 days | PND 3-8 | 30 min; 3x/day | Increased times that dams left the nest; no significant differences in the duration of the dam-pups interaction. | 12 |
| Hsiao (2016) | C57BL/6 | Yes (5 pups) | Yes | Limited Bedding | PND 2-9 | 8 days | PND 2-8 | 30 min (observed every 1 min); 2x/day | Increased number of exits from the nest; no significant differences in licking and grooming. | 11 |
| Lesuis (2018) | C57BL/6J | Yes (6 pups) | NR | Limited Bedding | PND 2-9 | 8 days | PND 3-8 | 48 min; 2x/day | Increased number of exits from the nest and pups out of nest; increased time off pups (light phase); decreased duration of nursing (light phase). | 11,5 |
| Luchetti (2015) | NMRI | Yes (8 pups) | NR | Handling / Repeated Cross-fostering | PND 1-14 / PND 1-4 | 15 min / 4 times | PND 2-7 | 32 min (observed every 2 min); 2x/day | Increased nursing (PND 2-4); no significant difference in licking and grooming. | 12,5 |
| Magalhães (2017) | C57BL/6 | NR | Yes | Sibling Separation | PND 5-21 | 6 hours | PND 5, 14 and 20 | 10 or 15 min; 2x/day | Increased nesting, nursing, breast feeding (after stress); decreased absence of contact (after stress); no significant difference in self-grooming, eating, drinking and resting. | 12 |
| Moles (2004) | NMRI | Yes (8 pups) | NR | Maternal Separation / Strange Male bedding | PND 1-14 | 15 min | PND 2, 4, 6, 8, 10 and 12 | 30 min (observed every 1 min); 1x/day | No significant differences in nursing, grooming, caring pup, nest building, exploration of the cage, feeding and self-grooming. | 12 |
| Orso (2017) | BALB/c | Yes (5-7 pups) | Yes | Maternal Separation | PND 2-7 | 3 hours | PND 2-7 | 15 min (observed every 3 min); 6x/day | Increased eating and drinking (PND 2); increased no interaction (PND 2 and 3); increased nursing (PND 7); decreased no interaction (PND 7); decreased nursing (PND 2 and 3); no significant differences in licking and contact with pups. | 12 |
| Own (2013) | C57BL/6 | Yes (6-8 pups) | NR | Maternal Separation | PND 2-14 | 3 hours / 15 min | PND 2, 5, 8, 11 and 14 | 2 hours (observed every 30 sec) pre-MS and 15 min (observed every 10 sec) post-MS | Increased pup handling, licking, nursing, covering and nesting (after stress). | 12 |
| Rice (2008) | C57BL/6J | Yes (5 pups) | NR | Limited Bedding | PND 2-9 | 8 days | PND 2-8 | 30 min (observed every 1 min); 3x/day | Increased number of exits from the nest; no significant differences in the duration of the dam in the nest. | 12,5 |
| Romeo (2003) | C57BL/6 | No | No | Maternal Separation | PND 3-14 | 3 hours | PND 1-14 | 30 min (observed every 1 min); 1x/day | No significant differences in arched-back nursing, nursing prone, nest building, licking, grooming and off the nest. | 10,5 |
| Wei (2010) | C57BL/6 and BALB/CBYJ | Yes (6-8 pups) | Yes | Maternal Separation | PND 1-21 | 40 min | PND 0-7 | 60 min (observed every 4 min); 4x/day | Increased licking, grooming and arched-back nursing (PND 1-7); decreased no contact with the pups (PND 1-7); no significant difference in time spent away from pups and passive nursing. | 12,5 |

*Note:* NR = Not Reported; PND = Postnatal Day.
