# Supplementary material for: How Early Life Stress Impact Maternal Care: A Systematic Review of Rodent Studies
Source: Front Behav Neurosci. 2019 Aug 28;13:197. doi: 10.3389/fnbeh.2019.00197 (PMC6724664; doi:10.3389/fnbeh.2019.00197)
Supplement: Supplementary file 3 [file Table_5.DOCX]

Supplementary Material


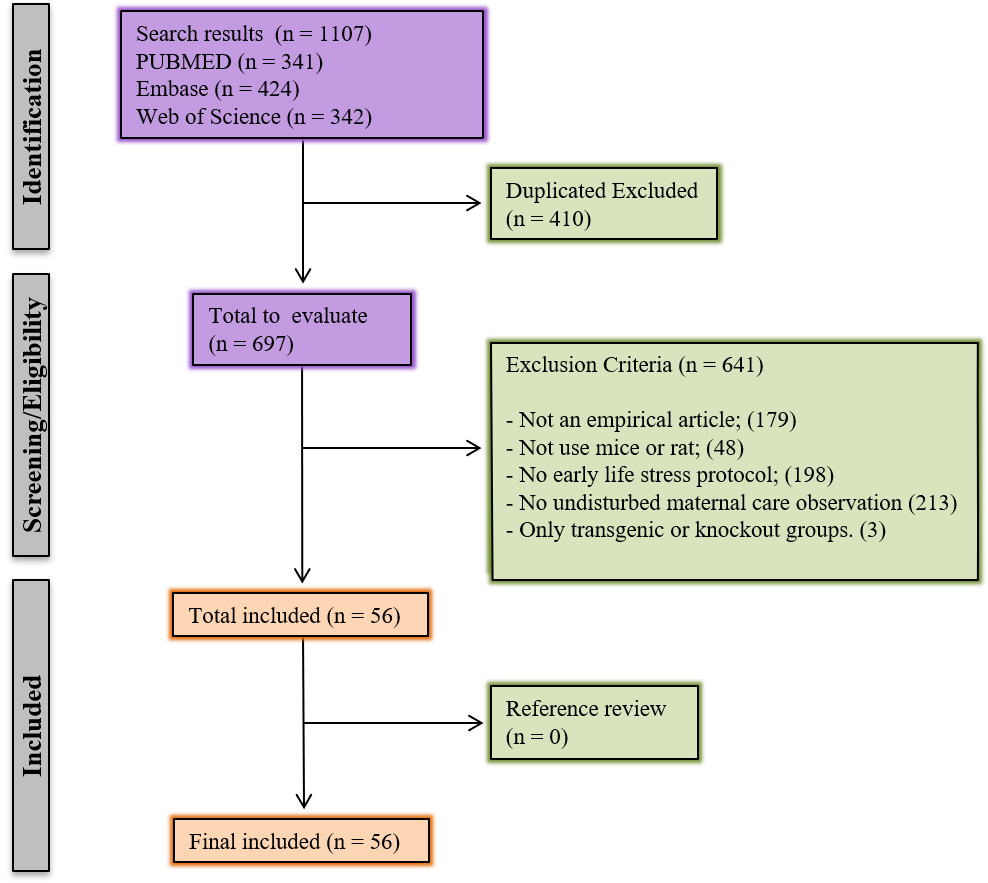


**Supplementary Figure 1.** Flow chart of the systematic review.



**Supplementary Figure 2.** Percentage of methodological report criteria fulfilled by rat studies.



**Supplementary Figure 3.** Percentage of methodological report criteria fulfilled by mice studies.


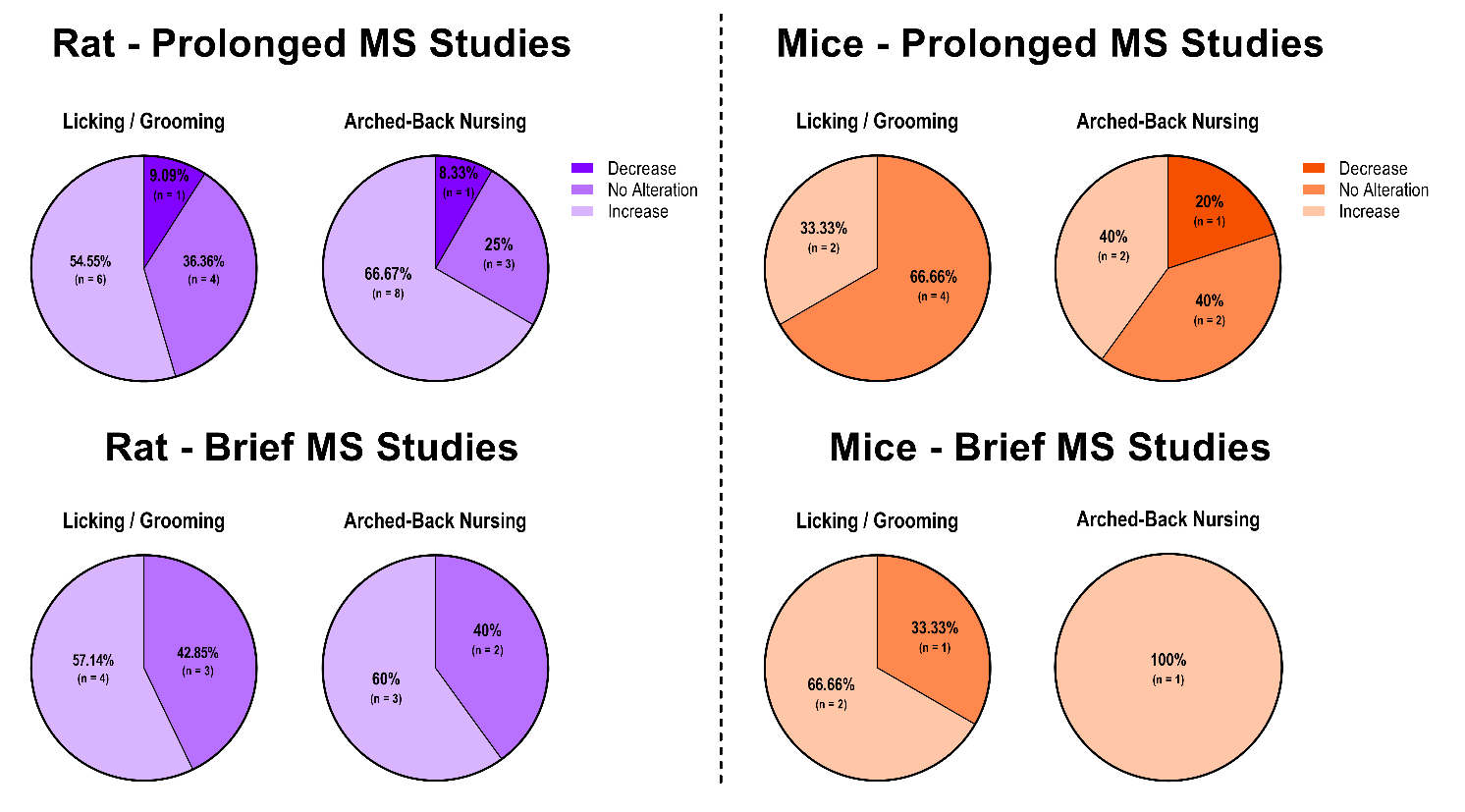
**Supplementary Figure 4.** Percentage of licking/grooming and arched-back nursing from prolonged and brief maternal separation studies.


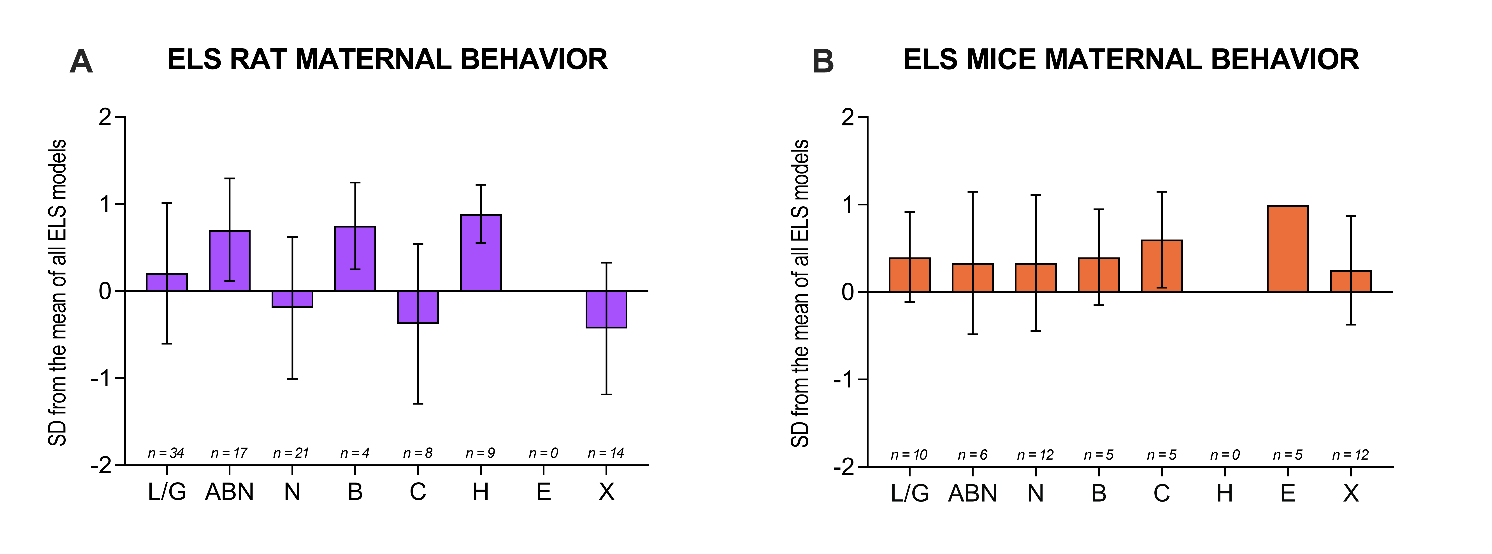
**Supplementary Figure 5.** Variability of the results from the overall ELS impact on maternal behavior. L/G = licking/grooming; ABN = arched-back nursing; N = passive/blanket nursing; B = nest building; C = contact with pups; H = harmful caregiving; E = nest exists; X = no contact. n = total number of evidences for each behavior. 1 = increase; 0 = no alteration; -1 = decrease. SD = Standard Deviation.
